# Mutations in *POGLUT1* in Galli–Galli/Dowling–Degos disease

**DOI:** 10.1111/bjd.14914

**Published:** 2016-09-24

**Authors:** N.J. Wilson, C. Cole, K. Kroboth, W.N. Hunter, J.A. Mann, W.H.I. McLean, K. Kernland Lang, H. Beltraminelli, R.A. Sabroe, N. Tiffin, G.J. Sobey, L. Borradori, E. Simpson, F.J.D. Smith

**Affiliations:** ^1^ Dermatology and Genetic MedicineDivision of Biological Chemistry and Drug DiscoverySchool of Life SciencesUniversity of DundeeDundeeU.K.; ^2^ Division of Computational BiologySchool of Life SciencesUniversity of DundeeDundeeU.K.; ^3^ Division of Biological Chemistry and Drug DiscoverySchool of Life SciencesUniversity of DundeeDundeeU.K.; ^4^ Department of DermatologyOregon Health & Sciences UniversityPortlandORU.S.A.; ^5^ Division of DermatologyDartmouth‐Hitchcock Medical CenterLebanonNHU.S.A.; ^6^ Department of DermatologyUniversity Hospital‐Inselspital BernUniversity of BernBernSwitzerland; ^7^ Department of DermatologyRoyal Hallamshire HospitalSheffieldU.K.; ^8^ Department of HistopathologyRoyal Hallamshire HospitalSheffieldU.K.; ^9^ Department of Clinical GeneticsSheffield Children's HospitalSheffieldU.K.; ^10^ Pachyonychia Congenita ProjectSalt Lake CityUTU.S.A.

dear editor, The group of reticulate pigmentary disorders includes the rare autosomal dominant Dowling–Degos disease (DDD) and Galli–Galli disease (GGD; OMIM 179850, 615327 and 615696).^1^ In light of substantial clinical, histological and mutational overlap between GGD and DDD, they are considered to belong to the same entity.^2^, ^3^ Mutations in *KRT5* (encoding keratin 5) have been associated with GGD/DDD since 2006.^2^, ^3^, ^4^, ^5^ With the development of whole‐exome sequencing, mutations in *POFUT1* (encoding protein *O*‐fucosyltransferase 1)^6^, ^7^ and *POGLUT1* (encoding protein *O*‐glucosyltransferase 1)^8^ have been shown to underlie some cases of GGD/DDD. We report mutations in *POGLUT1* in three families of European ancestry.

In family 1 the mother presented, aged 75 years, with a 40‐year history of reticulate hyperpigmented scaling plaques on the neck, lateral proximal arms, proximal legs and popliteal fossae (Fig. 1a, b). Light microscopy revealed epidermal acanthosis, hypergranulosis and hyperkeratosis with parakeratosis and suprabasilar acantholysis (Fig. 1c). Direct DNA sequencing of *KRT5* was negative for mutations. A whole‐exome sequencing approach was taken using DNA from the affected mother (Appendix S1 and Table S1; see Supporting Information). During this time mutations were reported in *POFUT1* and *POGLUT1*. A previously reported heterozygous nonsense mutation was identified in *POGLUT1*, p.Arg218*; c.652C>T, and confirmed by Sanger sequencing. The mutation was identified in her affected son but not in two unaffected daughters.

**Figure 1:**
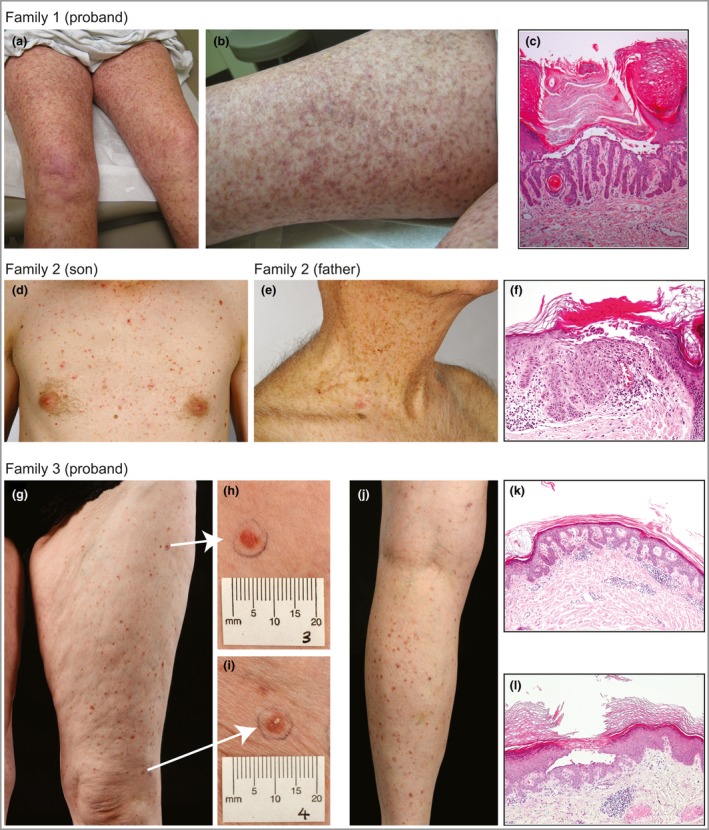
Clinical findings. (a, b) Reticulate hyperpigmented scaling plaques on the proximal legs of the proband of family 1. (c) Light microscopy of three punch biopsies from the proband of family 1 revealed similar findings of filiform digitate downgrowth of the epidermal rete ridges, presence of small horn cysts, epidermal acanthosis, hypergranulosis, and hyperkeratosis with parakeratosis and suprabasilar acantholysis. (d) Small red‐brown macules and crusted papules widely distributed over the trunk, neck, back, abdomen and limbs of the proband of family 2. The large body folds, hands and feet, and face were spared. (e) The father of the proband of family 2 had similar clinical features, limited to the neck region without involvement of the large flexures. (f) Light microscopy shows some apical acantholysis and parakeratosis with a moderate dermal inflammatory infiltrate. (g–j) The proband of family 3 with multiple small (approximately 5‐mm‐diameter) red‐brown scaly papules, present predominantly on the legs but also on the forearms with some postinflammatory change from previous lesions. (k) Light microscopy from the proband of family 3, a medium‐power view showing elongation of the rete ridge and modest inflammatory infiltrate. (l) A medium‐power view of a second biopsy from the right anterior thigh. There is an intraepidermal vesicle, as a result of acantholysis of the keratinocytes, some of which show dyskeratosis. Note the elongation of the rete ridges immediately below the vesicle.

A second family with GGD/DDD, present in two generations, was also negative for *KRT5* mutation. The proband was a 40‐year‐old man who progressively developed, starting from adolescence, small red‐brown macular and papular crusted lesions (Fig. 1d). His father had similar clinical features (Fig. 1e). Light microscopy showed filiform digitate downgrowth of epidermal rete ridges, presence of small horn cysts, apical acantholysis and parakeratosis with a moderate dermal inflammatory infiltrate (Fig. 1f). *POFUT1* and *POGLUT1* were screened by Sanger sequencing (Appendix S1). A previously unreported heterozygous missense mutation was identified in *POGLUT1*, p.Gly170Glu; c.509G>A, in the affected father and affected son, but not in the unaffected mother or unaffected brother (Fig. 2a, b). p.Gly170 is highly conserved in the encoded enzyme across species. *In silico* prediction tools, MutationTaster (http://www.mutationtaster.org/) and PolyPhen‐2 (http://genetics.bwh.harvard.edu/pph2/), predict this to be a disease‐causing variant. It is not in the dbSNP (http://www.ncbi.nlm.nih.gov/SNP/), NHLBI Exome Variant Server (http://evs.gs.washington.edu/EVS/) or ExAC (http://exac.broadinstitute.org/) databases. No mutations were identified in *POFUT1*.

**Figure 2:**
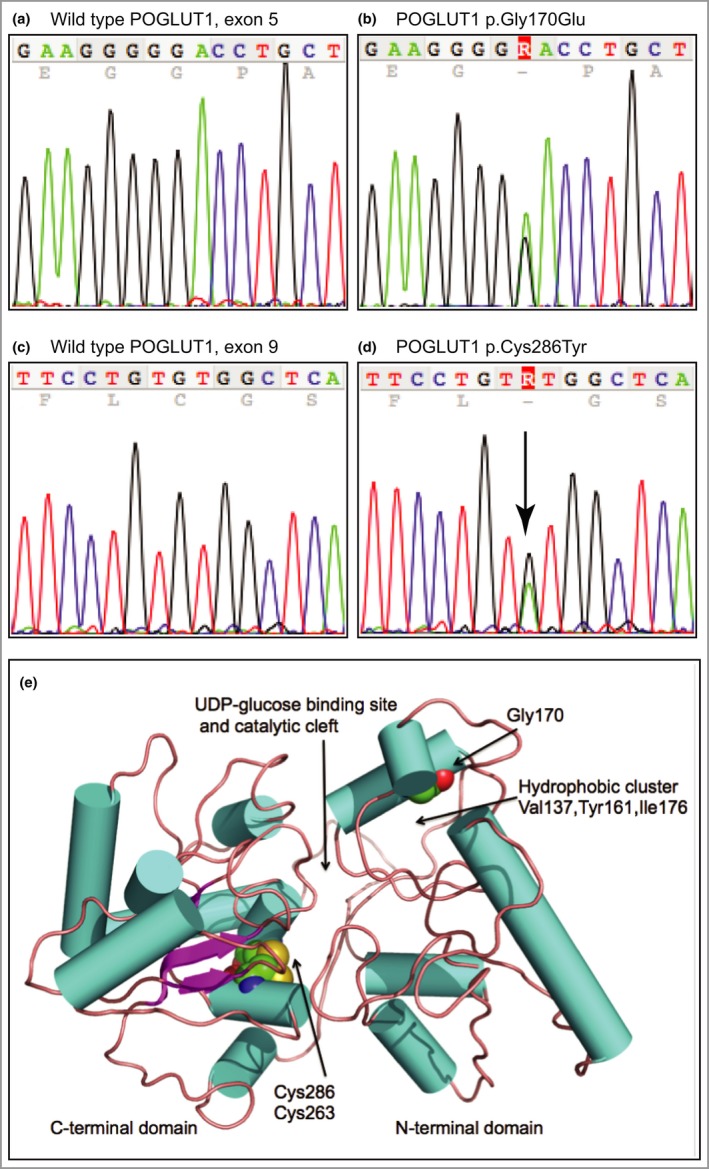
Mutation analysis and protein modelling. (a) Wild‐type *POGLUT1* sequence of exon 5 showing nucleotides c.502–516. (b) Equivalent region as in (a) from the proband of family 2, showing the heterozygous mutation c.509G>A leading to missense mutation p.Gly170Glu. (c) Wild‐type *POGLUT1* sequence showing nucleotides c.850–864 of exon 9. (d) Equivalent region as in (c) from the proband of family 3, showing the heterozygous mutation c.857G>A resulting in missense mutation p.Cys286Tyr. (e) The protein model of human POGLUT1. The enzyme structure is shown in cartoon format with cylinders (cyan) to represent α‐helices, and arrows (purple) β‐strands that are linked by coils (brown). The residues Gly170, Cys263 and Cys286 are depicted in CPK representation coloured N blue, S yellow, O red and C green. The *N*‐terminal domain consists of residues 1–180. The modelling predicts that each missense mutation, p.Gly170Glu and p.Cys286Tyr, would compromise enzyme activity due to a reduction in stability of the protein fold near the active site.

A 52‐year‐old woman, in family 3, presented at age 50 years with a 10‐year history of multiple, sometimes itchy, small red‐brown scaly lesions. These started on her legs then spread onto her forearms with a few lesions on her trunk (Fig. 1g–j). Her mother had similar lesions. Light microscopy of different skin biopsies variably showed focal hyperkeratosis, focal parakeratosis, acantholysis and elongation of rete ridges with a mild patchy chronic inflammatory infiltrate in the upper dermis (Fig. 1k). One biopsy showed acantholysis, (Fig. 1l). Screening of *KRT5* and *POFUT1* did not yield any mutations. A heterozygous missense mutation was identified in *POGLUT1*, p.Cys286Tyr; c.857G>A, in both affected individuals (Fig. 2c, d). PolyPhen‐2 and MutationTaster predict this previously unreported variant as disease causing; it is not in the dbSNP, NHLBI Exome Variant Server or ExAC databases. p.Cys286 is highly conserved across a number of species.

Mutations in *POGLUT1* were reported by Basmanav *et al*.^8^ in 13 unrelated individuals. The majority were nonsense mutations, but there were also a splice‐site and a missense mutation. We identified one previously reported nonsense mutation, p.Arg218*, and two previously unreported missense mutations, p.Gly170Glu and p.Cys286Tyr. The p.Arg218* mutation results in a truncated form of POGLUT1 that lacks part of the substrate‐binding site required for high‐affinity binding of UDP‐glucose.^8^

Using homology modelling, we investigated the effects of the two missense mutations (Fig. 2e). Gly170 is on a short helical segment with *C*‐alpha hydrogens directed into a hydrophobic pocket that likely stabilizes a subdomain, part of the *N*‐terminal domain. This pocket is about 15 Å distant from the site of catalysis. A mutation to glutamate places a polar and acidic side chain into this hydrophobic environment. The result would be potentially destabilizing electrostatic interactions and steric clashes with Val137, Tyr161 and Ile176. Cys286 is in the *C*‐terminal domain, close to Cys263. The model strongly suggests that a disulfide bond would be formed by these two residues. Mutation of Cys286 to tyrosine would remove the highly stabilizing disulfide bond that holds together distinct elements of secondary structure, and then, depending on which side‐chain rotamers were adopted, to clash with Phe278, His282, Leu283, Val260 and Ser288, which form part of the hydrophobic core of the *C*‐terminal domain. Again, the consequence would be to destabilize the protein fold at a position about 15 Å from the site of catalysis. These models predict that each missense mutation, p.Gly170Glu and p.Cys286Tyr, would compromise enzyme activity due to a reduction in stability of the protein fold near the active site.

POGLUT1 adds *O*‐linked glucose to serine residues in epidermal growth factor‐like repeats of Notch receptors, and is an essential regulator of Notch signalling. Loss of Notch1 and Notch2 in mice results in abnormal pigmentation; these and other studies suggest that mutations in *POGLUT1* resulting in aberrations in Notch signalling would lead to abnormal pigmentation and keratinocyte morphology.^9^, ^10^

Differences were noted between our cases. In family 2, lesions were widely scattered over the entire trunk and limbs (son) and on the neck and upper trunk region (father), rather than in a reticulated pattern. Similarly, in family 3 there was no reticulate rash compared with that of family 1. With only a small number of cases reported it is difficult to draw any genotype–phenotype correlation.

These findings expand the spectrum of mutations in *POGLUT1* and confirm *POGLUT1* as the third candidate gene, along with *KRT5* and *POFUT1*, to consider in diagnosis of GGD/DDD.

## Supporting information


**Appendix S1.** Supplementary methods.

**Table S1.** *POGLUT1* primers for polymerase chain reaction and sequencing.
