# Supplementary material for: Mutations in POGLUT1 in Galli–Galli/Dowling–Degos disease
Source: Br J Dermatol. 2016 Sep 24;176(1):270–4. doi: 10.1111/bjd.14914 (PMC5324688; doi:10.1111/bjd.14914)
Supplement: Supplementary file 1 — Appendix S1. Supplementary methods. [file BJD-176-270-s001.docx]

**Supplementary material**

Methods

Genomic DNA was extracted from peripheral blood leukocytes using standard procedures. Samples were obtained with informed consent and ethical approval by a by the Oregon Health and Sciences University Institutional Review Board (Family 1) and by a Western Institutional Review Board (Families 2 and 3) that complies with principles of the Helsinki Accord.

WES was performed with Agilent SureSelect v4 capture kit and sequenced on an Illumina HiSeq 2000 (Edinburgh Genomics, Edinburgh). The resulting 50.1M 100 bp PE sequencing reads were aligned to the human genome (Ensembl release 68) with Bowtie2 (v2.02) (98.2% aligned) (Langmead and Salzberg, 2012). Read duplicates were removed with Picard Tools (v1.79) (<http://picard.sourceforge.net>) and variants were called using the UnifiedGenotyper in GATK-lite (v2.2-8) following the GATK ‘best practices’ (McKenna *et al.*, 2010). The 57,788 called variants were annotated with the Variant Effect Predictor v72 (McLaren *et al.*, 2010) and any variants with the following consequences were filtered out: downstream_gene_variant, upstream_gene_variant, synonymous_variant, intergenic_variant and intron_variant. Filtered data were put in a MySQL database allowing querying via a Django interface. The data was mined initially for mutations in the candidate genes, *KRT5, POFUT1* and *POGLUT1*.

The coding region and intron/exon boundaries of *POGLUT1* were amplified using primers specific to *POGLUT1* (NM_152305), Supplementary table 1. Primers were checked for SNPs using Diagnostic SNPCheck (www.ngrl.org.uk/Manchester). PCR reactions were set up in Qiagen Coral Load PCR buffer containing 1.5mM MgCl_2_ and 1U HotStarTaq *Plus* DNA Polymerase (Qiagen, Crawley, UK). The following PCR conditions were used (95°C 5 min) x1; (94°C 1 min, 60°C 1 min, 72°C 1 min) x 35; and (72°C 10 min) x 1. PCR products were purified using QiaQuick PCR spin columns (Qiagen, Crawley, UK) and sequenced on an ABI 3730 Automated DNA sequencing machine (Foster City, CA) according to the manufacturer’s instructions. The frequency of each sequence change was checked on the dbSNP database and Exome Variant Server (http://evs.gs.washington.edu/EVS).

Homology modeling of human POGLUT1 was carried out in similar fashion to that reported by Basmanav *et al*. (2104) where the ModBase resource (Pieper *et al.*, 2014) provided the initial model. As an independent check and for comparison another model was prepared using PHYRE2 (Kelley *et al*., 2015). The computer graphics program COOT (Emsley & Cowtan, 2004) was used for model inspections, then mutagenesis, and visualization of different side chain rotamers using the ModBase model. PYMOL (Schrödinger, 2010) was used to prepare the molecular figure.

**References for supplementary methods**

Emsley P and Cowtan K. Coot: model-building tools for molecular graphics. *Acta Crystallogr. D Biol. Crystallogr*. 2004 **60**:2126–2132.

Kelley LA, Mezulis S, Yates CM, *et al.* The Phyre2 webportal

for protein modeling, prediction and analysis. *Nat. Protoc*. 2015 **10:**845-58.

Langmead B and Salzberg SL*,* Fast gapped-read alignment with Bowtie 2. *Nat Methods* 2012;**9**:357-9.

McKenna A, Hanna M, Banks E*, et al.* The Genome Analysis Toolkit: a MapReduce framework for analyzing next-generation DNA sequencing data. *Genome Research* 2010*;***20**:1297-303.

McLaren W, Pritchard B, Rios D*, et al.* Deriving the consequences of genomic variants with the Ensembl API and SNP Effect Predictor. *Bioinformatics* 2010*;***26**:2069-70.

Pieper U, Webb BM, Dong GQ, *et al.* ModBase, a database

of annotated comparative protein structure models and associated resources.

*Nucleic Acids Res*. 2014 **42**:D336-46.

Schrödinger, L.L.C. (2010). The PyMOL Molecular Graphics System.
