# Supplementary material for: Mutations in POGLUT1 in Galli–Galli/Dowling–Degos disease
Source: Br J Dermatol. 2016 Sep 24;176(1):270–4. doi: 10.1111/bjd.14914 (PMC5324688; doi:10.1111/bjd.14914)
Supplement: Supplementary file 2 — Table S1. POGLUT1 primers for polymerase chain reaction and sequencing. [file BJD-176-270-s002.docx]

**Supplementary Table 1 *POGLUT1* primers for PCR and sequencing**

| Exon | *POGLUT1* Primers | Size (bp) |
| --- | --- | --- |
| 1 | Ex1F 5’ ACGGTGGCCATCTTTGTG 3’ | 324 |
|  | Ex1R 5’ GCTCCGAGCTACAGCGG 3’ |  |
| 2 | Ex2F 5’ TCTGTTGGTGTGGTGTCAGC 3’ | 231 |
|  | Ex2R 5’ AAGGATGTTAGAATACTTCCTGTGAC 3’ |  |
| 3 | Ex3F 5’ GGAATGGCACCACTCTCTTG 3’ | 284 |
|  | Ex3R 5’ GTCCCATGAATTGGCTCCTC 3’ |  |
| 4 | Ex4F 5’ ACCTCGCCTTGTCCTAGAGC 3’ | 271 |
|  | Ex4R 5’ GACAAAGATCTCATGCTCATATCC 3’ |  |
| 5 | Ex5F 5’ AAGGCTGTCCAGATCCTGTG 3’ | 325 |
|  | Ex5R 5’ AAAGGGCGAAACTCCGTC 3’ |  |
| 6 | Ex6F 5’ AATCTTCAGAAATGGTTAAAGCAC 3’ | 285 |
|  | Ex6R 5’ CTCGAATCTAAAATTGTGATTCC 3’ |  |
| 7 | Ex7F 5’ ACCTTTCAGGGATTGGGAAC 3’ | 236 |
|  | Ex7R 5’ AGGTGGCAATGTTCTTTGAG 3’ |  |
| 8 | Ex8F 5’ TTTAACAATGCTTGCTGCAC 3’ | 247 |
|  | Ex8R 5’ GCAACTTTCATTTACTCCAGC 3’ |  |
| 9 | Ex9F 5’ CCCAGAACAACCTGTAGTCCTAG 3’ | 433 |
|  | Ex9R 5’ TTTTAAAAGCAGATTGGAAGGG 3’ |  |
| 10 | Ex10F 5’ CTGACTTAGTGCCAATGAAGTTG 3’ | 265 |
|  | Ex10R 5’ CCCAGGACAGGTTTAGTTCTG 3’ |  |
| 11 | Ex11F 5’ TGCTGAATAATGCTTGGCAC 3’ | 334 |
|  | Ex11R 5’ CAAGCTTATGGTAAGCTTCTCAC 3’ |  |
